# Peer Review of “Machine Learning–Based Hyperglycemia Prediction: Enhancing Risk Assessment in a Cohort of Undiagnosed Individuals”

**DOI:** 10.2196/60393

**Published:** 2024-09-11

**Authors:** Tarek Abd El-Hafeez

**Affiliations:** 1 Deraya University, Deraya Square, New Minia, Egypt

**Keywords:** hyperglycemia, diabetes, machine learning, hypertension, random forest

*This is the peer-review report for “Machine Learning–Based Hyperglycemia Prediction: Enhancing Risk Assessment in a Cohort of Undiagnosed Individuals.”*

## Round 1 Review

1. In this paper [1], describe dataset features in more detail and its total size and size (train/test) as a table.
2. Pseudocode/flowchart and algorithm steps need to be inserted.
3. Time spent needs to be measured in the experimental results.
4. Limitation and Discussion sections need to be inserted.
5. All metrics need to be calculated such as precision, recall, and receiver operating characteristic curves in the experimental results.
6. The parameters used for the analysis must be provided in a table.
7. The architecture of the proposed model must be provided.
8. The authors need to make a clear proofread to avoid grammatical mistakes and typo errors.
9. Add future work in last section (conclusion), if any.
10. The authors need to add recent articles in related work and update them.
11. To improve the Related Work and Introduction sections, authors are recommended to review these highly related research work papers:
  - El-Hafeez TA, Shams MY, Elshaier YAMM, Farghaly HM, Hassanien AE. Harnessing machine learning to find synergistic combinations for FDA-approved cancer drugs. *Sci Rep*. Jan 29, 2024;14(1):2428. [doi: 10.1038/s41598-024-52814-w] [Medline: 38287066]
  - Hassan E, El-Hafeez TA, Shams MY. Optimizing classification of diseases through language model analysis of symptoms. *Sci Rep*. Jan 17, 2024;14(1):1507. [doi: 10.1038/s41598-024-51615-5] [Medline: 38233458]
  - Omar A, El-Hafeez TA. Optimizing epileptic seizure recognition performance with feature scaling and dropout layers. *Neural Computing Applications*. Nov 24, 2024;36:2835-2852. [doi: 10.1007/s00521-023-09204-6]
  - Hady DAA, El-Hafeez TA. Predicting female pelvic tilt and lumbar angle using machine learning in case of urinary incontinence and sexual dysfunction. *Sci Rep*. Oct 20, 2023;13(1):17940. [doi: 10.1038/s41598-023-44964-0] [Medline: 37863988]
  - Eliwa EHI, El Koshiry AM, El-Hafeez TA, Farghaly HM. Utilizing convolutional neural networks to classify monkeypox skin lesions. *Sci Rep*. Sep 3, 2023;13(1):14495. [doi: 10.1038/s41598-023-41545-z] [Medline: 37661211]
  - Farghaly HM, Shams MY, El-Hafeez TA. Hepatitis C Virus prediction based on machine learning framework: a real-world case study in Egypt. *Knowledge Inf Syst*. Mar 2, 2023;65:2595-2617. [doi: 10.1007/s10115-023-01851-4]

## Round 2 Review

Accept.
